# Supplementary material for: Modeling glioblastoma heterogeneity as a dynamic network of cell states
Source: Mol Syst Biol. 2021 Sep 16;17(9):e10105. doi: 10.15252/msb.202010105 (PMC8444284; doi:10.15252/msb.202010105)
Supplement: Supplementary file 5 — Source Data for Figure 3 [file MSB-17-e10105-s001.zip › Figure3A_sourcedata/GSEA_3065/nosigdb_state6.GseaPreranked.1622718443993/LARSSON_STATE_2.html]

Details for gene set LARSSON\_STATE\_2[GSEA]

|  || Dataset | state6 |
| Phenotype | NoPhenotypeAvailable |
| Upregulated in class | na\_neg |
| GeneSet | LARSSON\_STATE\_2 |
| Enrichment Score (ES) | -0.8711208 |
| Normalized Enrichment Score (NES) | -3.3983288 |
| Nominal p-value | 0.0 |
| FDR q-value | 0.0 |
| FWER p-Value | 0.0 |
Table: GSEA Results Summary

  

Fig 1: Enrichment plot: LARSSON\_STATE\_2      
 Profile of the Running ES Score & Positions of GeneSet Members on the Rank Ordered List

  

| PROBE | GENE SYMBOL | GENE\_TITLE | RANK IN GENE LIST | RANK METRIC SCORE | RUNNING ES | CORE ENRICHMENT || 1 | ADGRG1 |  |  | 4417 | -0.007 | -0.5066 | No |
| 2 | MIAT |  |  | 5393 | -0.026 | -0.6171 | No |
| 3 | SCRG1 |  |  | 5450 | -0.027 | -0.6221 | No |
| 4 | MTRNR2L12 |  |  | 6287 | -0.044 | -0.7157 | No |
| 5 | NCAM1 |  |  | 6342 | -0.046 | -0.7195 | No |
| 6 | LAPTM4B |  |  | 6862 | -0.061 | -0.7758 | No |
| 7 | BCAN |  |  | 6866 | -0.061 | -0.7729 | No |
| 8 | FGFBP3 |  |  | 7004 | -0.065 | -0.7852 | No |
| 9 | GRIA2 |  |  | 7312 | -0.075 | -0.8165 | No |
| 10 | CREBZF |  |  | 7316 | -0.075 | -0.8128 | No |
| 11 | NOVA1 |  |  | 7506 | -0.082 | -0.8302 | No |
| 12 | SOX6 |  |  | 7616 | -0.087 | -0.8380 | No |
| 13 | DDR1 |  |  | 7836 | -0.098 | -0.8580 | No |
| 14 | ANKRD11 |  |  | 7843 | -0.098 | -0.8534 | No |
| 15 | GPM6A |  |  | 7875 | -0.100 | -0.8517 | No |
| 16 | FTX |  |  | 8004 | -0.107 | -0.8606 | No |
| 17 | MARCH6 |  |  | 8038 | -0.109 | -0.8586 | No |
| 18 | SPPL2B |  |  | 8148 | -0.117 | -0.8649 | Yes |
| 19 | PON2 |  |  | 8177 | -0.120 | -0.8617 | Yes |
| 20 | DDX39B |  |  | 8243 | -0.125 | -0.8625 | Yes |
| 21 | TMEM259 |  |  | 8275 | -0.129 | -0.8592 | Yes |
| 22 | ACSL3 |  |  | 8295 | -0.132 | -0.8544 | Yes |
| 23 | WSB1 |  |  | 8366 | -0.142 | -0.8549 | Yes |
| 24 | LAMP2 |  |  | 8425 | -0.149 | -0.8536 | Yes |
| 25 | PCSK7 |  |  | 8446 | -0.152 | -0.8478 | Yes |
| 26 | RCN2 |  |  | 8451 | -0.152 | -0.8401 | Yes |
| 27 | GPC1 |  |  | 8473 | -0.155 | -0.8343 | Yes |
| 28 | ZNF708 |  |  | 8497 | -0.161 | -0.8283 | Yes |
| 29 | LRPAP1 |  |  | 8498 | -0.161 | -0.8198 | Yes |
| 30 | MSMO1 |  |  | 8505 | -0.162 | -0.8118 | Yes |
| 31 | ZNF431 |  |  | 8520 | -0.166 | -0.8046 | Yes |
| 32 | COL4A2 |  |  | 8525 | -0.168 | -0.7961 | Yes |
| 33 | MIR99AHG |  |  | 8543 | -0.171 | -0.7890 | Yes |
| 34 | PGRMC1 |  |  | 8555 | -0.174 | -0.7810 | Yes |
| 35 | HACD3 |  |  | 8558 | -0.175 | -0.7719 | Yes |
| 36 | SF1 |  |  | 8565 | -0.177 | -0.7632 | Yes |
| 37 | ATP6AP2 |  |  | 8607 | -0.195 | -0.7575 | Yes |
| 38 | DDX17 |  |  | 8613 | -0.196 | -0.7476 | Yes |
| 39 | LUC7L3 |  |  | 8631 | -0.203 | -0.7388 | Yes |
| 40 | GPM6B |  |  | 8632 | -0.203 | -0.7279 | Yes |
| 41 | TTC14 |  |  | 8635 | -0.205 | -0.7172 | Yes |
| 42 | PDIA3 |  |  | 8647 | -0.212 | -0.7072 | Yes |
| 43 | PRDX4 |  |  | 8660 | -0.217 | -0.6970 | Yes |
| 44 | COL4A1 |  |  | 8661 | -0.218 | -0.6854 | Yes |
| 45 | XRCC2 |  |  | 8662 | -0.218 | -0.6738 | Yes |
| 46 | KANSL1 |  |  | 8669 | -0.223 | -0.6626 | Yes |
| 47 | TMBIM6 |  |  | 8673 | -0.225 | -0.6510 | Yes |
| 48 | SSR4 |  |  | 8689 | -0.233 | -0.6403 | Yes |
| 49 | ARGLU1 |  |  | 8690 | -0.234 | -0.6279 | Yes |
| 50 | ZNF75A |  |  | 8701 | -0.239 | -0.6163 | Yes |
| 51 | DDOST |  |  | 8706 | -0.242 | -0.6039 | Yes |
| 52 | SNTG1 |  |  | 8713 | -0.251 | -0.5912 | Yes |
| 53 | B2M |  |  | 8718 | -0.258 | -0.5780 | Yes |
| 54 | PRH1 |  |  | 8722 | -0.260 | -0.5644 | Yes |
| 55 | LAPTM4A |  |  | 8730 | -0.267 | -0.5510 | Yes |
| 56 | FUS |  |  | 8731 | -0.268 | -0.5368 | Yes |
| 57 | CANX |  |  | 8735 | -0.270 | -0.5227 | Yes |
| 58 | KCNQ1OT1 |  |  | 8742 | -0.281 | -0.5085 | Yes |
| 59 | ADGRV1 |  |  | 8747 | -0.285 | -0.4937 | Yes |
| 60 | LENG8 |  |  | 8750 | -0.289 | -0.4786 | Yes |
| 61 | MDM4 |  |  | 8753 | -0.290 | -0.4634 | Yes |
| 62 | APLP2 |  |  | 8755 | -0.292 | -0.4480 | Yes |
| 63 | APP |  |  | 8758 | -0.300 | -0.4322 | Yes |
| 64 | HMGCR |  |  | 8759 | -0.301 | -0.4162 | Yes |
| 65 | VMP1 |  |  | 8760 | -0.302 | -0.4001 | Yes |
| 66 | CD9 |  |  | 8762 | -0.303 | -0.3841 | Yes |
| 67 | SPCS2 |  |  | 8763 | -0.307 | -0.3678 | Yes |
| 68 | NKTR |  |  | 8766 | -0.315 | -0.3512 | Yes |
| 69 | SQLE |  |  | 8767 | -0.319 | -0.3342 | Yes |
| 70 | PLAT |  |  | 8772 | -0.327 | -0.3172 | Yes |
| 71 | CALR |  |  | 8773 | -0.330 | -0.2997 | Yes |
| 72 | SGCE |  |  | 8774 | -0.333 | -0.2819 | Yes |
| 73 | NEAT1 |  |  | 8775 | -0.334 | -0.2641 | Yes |
| 74 | CD63 |  |  | 8776 | -0.335 | -0.2463 | Yes |
| 75 | PTPRZ1 |  |  | 8783 | -0.360 | -0.2279 | Yes |
| 76 | CALU |  |  | 8784 | -0.362 | -0.2086 | Yes |
| 77 | HSPA5 |  |  | 8785 | -0.369 | -0.1889 | Yes |
| 78 | MEG3 |  |  | 8786 | -0.370 | -0.1692 | Yes |
| 79 | TNC |  |  | 8787 | -0.389 | -0.1485 | Yes |
| 80 | SLC1A3 |  |  | 8788 | -0.413 | -0.1265 | Yes |
| 81 | PTN |  |  | 8789 | -0.426 | -0.1038 | Yes |
| 82 | LMAN1 |  |  | 8790 | -0.441 | -0.0803 | Yes |
| 83 | PDIA6 |  |  | 8792 | -0.448 | -0.0566 | Yes |
| 84 | HSP90B1 |  |  | 8794 | -0.487 | -0.0308 | Yes |
| 85 | MALAT1 |  |  | 8796 | -0.581 | -0.0000 | Yes |
Table: GSEA details [plain text format]

  

Fig 2: LARSSON\_STATE\_2: Random ES distribution      
 Gene set null distribution of ES for **LARSSON\_STATE\_2**
